# Novel *Kras*-mutant murine models of non-small cell lung cancer possessing co-occurring oncogenic mutations and increased tumor mutational burden

**DOI:** 10.1007/s00262-020-02837-9

**Published:** 2021-01-28

**Authors:** Ramin Salehi-Rad, Rui Li, Linh M. Tran, Raymond J. Lim, Jensen Abascal, Milica Momcilovic, Stacy J. Park, Stephanie L. Ong, Maryam Shabihkhani, Zi Ling Huang, Manash Paul, David B. Shackelford, Kostyantyn Krysan, Bin Liu, Steven M. Dubinett

**Affiliations:** 1 Department of Medicine, Division of Pulmonary and Critical Care, David Geffen School of Medicine at UCLA, 10833 Le Conte Avenue, 43-229 CHS, Los Angeles, CA 90095-1690, USA; 2 Department of Medicine, VA Greater Los Angeles Healthcare System, 11301 Wilshire Boulevard, Los Angeles, CA 90073, USA; 3 Department of Molecular and Medical Pharmacology, David Geffen School of Medicine at UCLA, 650 Charles E. Young Drive South, 23-120 CHS, Box 951735, Los Angeles, CA 90095-1735, USA; 4 Department of Pathology and Laboratory Medicine, David Geffen School of Medicine at UCLA, 757 Westwood Plaza, Los Angeles, CA 90095, USA; 5 Jonsson Comprehensive Cancer Center, UCLA, 8-684 Factor Building, Box 951781, Los Angeles, CA 90095-1781, USA

**Keywords:** Mouse cancer models, NSCLC, TMB, KRAS, LKB1, Immunotherapy

## Abstract

Conditional genetically engineered mouse models (GEMMs) of non-small cell lung cancer (NSCLC) harbor common oncogenic driver mutations of the disease, but in contrast to human NSCLC these models possess low tumor mutational burden (TMB). As a result, these models often lack tumor antigens that can elicit host adaptive immune responses, which limits their utility in immunotherapy studies. Here, we establish *Kras*-mutant murine models of NSCLC bearing the common driver mutations associated with the disease and increased TMB, by in vitro exposure of cell lines derived from GEMMs of NSCLC [*Kras*^*G12D*^ (K), *Kras*^*G12D*^*Tp53*^−/−^(KP), *Kras*^*G12D*^*Tp53*^+/−^*Lkb1*^−/−^ (KPL)] to the alkylating agent *N*-methyl-*N*-nitrosourea (MNU). Increasing the TMB enhanced host anti-tumor T cell responses and improved anti-PD-1 efficacy in syngeneic models across all genetic backgrounds. However, limited anti-PD-1 efficacy was observed in the KPL cell lines with increased TMB, which possessed a distinct immunosuppressed tumor microenvironment (TME) primarily composed of granulocytic myeloid-derived suppressor cells (G-MDSCs). This KPL phenotype is consistent with findings in human *KRAS*-mutant NSCLC where LKB1 loss is a driver of primary resistance to PD-1 blockade. In summary, these novel *Kras*-mutant NSCLC murine models with known driver mutations and increased TMB have distinct TMEs and recapitulate the therapeutic vulnerabilities of human NSCLC. We anticipate that these immunogenic models will facilitate the development of innovative immunotherapies in NSCLC.

## Introduction

Immune checkpoint inhibitors (ICIs) targeting the PD-1/PD-L1 axis have resulted in durable clinical responses and improved survival in NSCLC [1]. However, most patients either do not respond to treatment or develop resistance to therapy after an initial response. Favorable responses to ICIs are associated with high TMB, preexisting intratumoral CD8^+^ T cells, and high baseline PD-L1 expression within the TME [2–4]. An increased number of candidate MHC class I tumor-neoantigens and a clonal neoantigen burden have also been associated with improved responses to ICIs in NSCLC [5, 6]. These results support the hypothesis that the non-synonymous somatic mutations associated with increased TMB generate tumor-neoantigens which can be recognized by host T cells as non-self. Treatment with ICIs can stimulate neoantigen-specific T cells to mediate tumor regression.

While ICIs have transformed the treatment landscape of NSCLC, a key impediment to progress in the field of lung cancer immunotherapy is the lack of preclinical models that recapitulate the complexity of human NSCLC. Human NSCLCs have among the highest mutational burden of all malignancies, and commonly possess driver mutations that confer distinct phenotypes [7]. *KRAS* mutations are the most prevalent oncogenic drivers in NSCLC and frequently co-occur with mutations in *TP53* and *LKB1,* which define subgroups of patients with distinct biology and therapeutic vulnerabilities [8]. Although conditional GEMMs of *Kras*-mutant NSCLC possess the common driver mutations, studies reveal that these models harbor low TMB with few protein-altering mutations [9–11]. As a result, these GEMMs of NSCLC have limited value in the evaluation of host anti-tumor immune responses in translational preclinical immunotherapy studies [12]. To our knowledge, there are no reported murine models that possess both the critical driver mutations and relevant TMB observed in human NSCLC.

Herein, we report novel immunogenic *Kras*-mutant murine models of NSCLC bearing the common oncogenic mutations of the disease and increased TMB. These models possess unique TMEs and recapitulate the therapeutic vulnerabilities observed in human disease. Specifically, the cell line with LKB1-deficiency and increased TMB showed limited responses to anti-PD-1 therapy and therefore represents a clinically relevant preclinical model of NSCLC that is resistant to ICIs.

## Materials and methods

### Murine cell lines

Murine cell lines 1969B (labeled as the ‘KP’ cell line in the remainder of the manuscript), KP-2042, KP-1.1, and KP-3.1 were established from lung adenocarcinomas of conditional cre-lox-cre *Kras*^*G12D*^*Tp53*^−/−^*Luc* FVB mice that express firefly luciferase (Supplemental Figure S1A). Murine cell lines 1940A (labeled as the ‘KPL’ cell line in the remainder of the manuscript), KPL-1950A, KPL-1942B, and KPL-2 were established from *Kras*^*G12D*^*Tp53*^+/−^*Lkb1*^−/−^*Luc* (KPL) FVB mice. Whole exome sequencing (WES) analysis revealed that KPL cells lost the other allele of *Tp53* upon in vitro culture and, therefore, bear a *Kras*^*G12D*^*Tp53*^−/−^*Lkb1*^−/−^*Luc* genotype. The *Kras*^*G12D*^ LKR-13 line (labeled as the ‘K’ cell line in the remainder of the manuscript), established from a lung adenocarcinoma tumor from a *K-ras*^*LA1*^ mouse, was generously provided by Dr. Jonathan Kurie [13]. Each cell line was maintained in culture media (RPMI-1640 medium supplemented with 10% FBS and 1% penicillin/streptomycin) at 37 °C in a humidified atmosphere of 5% CO_2_.

### In vivo studies

FVB and 129-E mice were purchased from Charles River Laboratories. Tumor cells were implanted in 7–9-week-old mice subcutaneously at optimal doses as indicated in figure legends. Tumor length and width were measured by caliper and the volume calculated by the equation: 0.4 × length × width^2^. For bioluminescence studies, images were obtained with the IVIS Spectrum imager after intraperitoneal (IP) injection of D-luciferin (150 mg/kg). For immunotherapy studies, mice bearing ∼ 50 mm^3^ tumors were randomized and treated with 200 μg of anti-PD-1 antibody (BioXcell, Clone RMP1–14) or isotype control via IP injections three times weekly for four doses. Mice were housed in pathogen-free facilities at UCLA and all procedures were approved by the UCLA Animal Research Committee.

### Chemical treatment

Cells were seeded in T25 flasks and when ∼ 70% confluency was achieved, culture media was removed and the cells exposed to 100 μg/mL of MNU (Chem Service, NG-17031) in PBS for 45 min. After the removal of MNU, cells were washed with PBS twice and fresh culture media was added. Cells were passaged a minimum of three times prior to the subsequent MNU exposure for up to seven cycles.

### In vitro proliferation assay

Cells were plated in culture media in 96-well plates at 1000 cells per well in eight replicates. Proliferation was measured using ATPlite 1step Luminescence Assay Kit (Perkin Elmer) every 24 h up to 120 h. Reading at each time point was normalized to the reading at baseline to control for plating differences.

### In vitro IFN-γ stimulation

Cells were seeded in 6-well plates and treated with IFN-γ at 100 ng/mL when 50% confluency was achieved. Cells were harvested 24 h after stimulation and PD-L1 expression was analyzed by flow cytometry (FACS).

### Tissue preparation

Spleens were mashed with the blunt end of 3 mL syringe on Petri dishes containing 5 mL of PBS, filtered through 70 μM filter, and centrifuged at 1500 rpm for 5 min at 4 °C. Cell pellets were resuspended in 5 mL of red blood cell lysis solution (BioLegend) on ice for 5 min followed by the addition of 20 mL of complete media. Cells were filtered through 70 μM filter, centrifuged, washed with PBS, and counted. Murine tumors were harvested, minced with scalpel blades, and digested in 2.5 mL of culture media containing 1 mg/mL of Collagenase IV (Roche) and 50 unit/mL DNase (Sigma) in 15 mL tubes at 37 °C with shaking every 10 min. After 45 min of incubation, 10 mL of fresh culture media was added and the samples were filtered through 70 μM filter and centrifuged. Red blood cells were lysed as described above, and the cells were washed with PBS, and counted.

### FACS

Single-cell suspension from tumor, spleen, or cell culture were incubated with antibodies for 20 min at 4 °C followed by washing with staining buffer (PBS + 2% FBS). Intracellular staining was performed using an eBioscience intracellular kit according to the manufacturer’s protocol. FACS was performed on Attune NxT cytometer (ThermoFisher), and data analyzed by FlowJo software (TreeStar). Details of the flow antibodies utilized are listed in the Supplemental Table S1.

### Genomic profiling

#### Genomic DNA isolation, library preparation, and sequencing

Genomic DNA was extracted from tumor cells (Qiagen, DNeasy blood and tissue kit) for WES. Tail DNA from three FVB and three 129-E mice was included as a normal reference for variant calls. Libraries for WES were prepared using the KAPA Hyper Prep Kit (Roche, KK8504) followed by exome enrichment with SeqCap EZ Share Developer Probe (Roche, 08333025001). Sequencing was performed on Hiseq3000 instrument as 150 bp pair-end runs with the aim of 100 × depth at UCLA TCGB Core facility.

#### Sequencing alignment

Sequence reads were aligned to the mouse genome (mm10) with Burrows-Wheeler Aligner (v 0.7.17), then marked for duplicates and re-calibrated as suggested by Genome Analysis Toolkit (GATK).

#### Variant calling and annotation

Strelka2 was utilized to call variants between cell line and the associated normal tail genome. When more than one reference normal genome was available (e.g. FVB mice), variant calls were performed to the individual normal genome independently and kept for down-stream analyses if they were called based on both reference genomes. Finally, a variant was called a mutation if (1) it was not in the germline mutation panel, determined from the FVB and 129-E tail genomes, (2) it was not supported by any read in the associated normal genome, (3) it was detected by at least 5 reads in cell lines, and (4) its variant allelic frequency (VAF) was > 0.1. The variants that passed these criteria were then annotated by Ensembl Variant Effect Predictor as non-synonymous mutations. Mutation with VAF ≥ 0.4 in all related cell lines including the parental cell line were defined as truncal. Shared mutations among members of the family with VAF < 0.4 were named as branch. Mutations with VAF < 0.4 which were specific to one cell line and not shared with the related cell lines were defined as private.

### Neoantigen prediction

Mutations altering amino acid sequences of the encoded proteins were subjected to MHC-I binding prediction utilizing NetH2pan [14]. Peptides subjected to the algorithm were composed of 8–11 amino acid residues with the mutated amino acid at different locations. A candidate neoantigen had its affinity score ranked < 2nd percentile based on the binding software recommendation.

### Statistical analysis

Statistical analyses were performed by GraphPad Prism version 7.04 for Windows. In brief, statistical significance was based on two-tailed non-paired Student’s *t* test for pairwise comparison and two-way ANOVA with Tukey post-test for time-associated comparison among multiple groups. Numerical data was presented as mean ± SEM.

## Results and discussions

### Establishing mouse models of NSCLC with known driver mutations and varying TMB

**Fig. 1:**
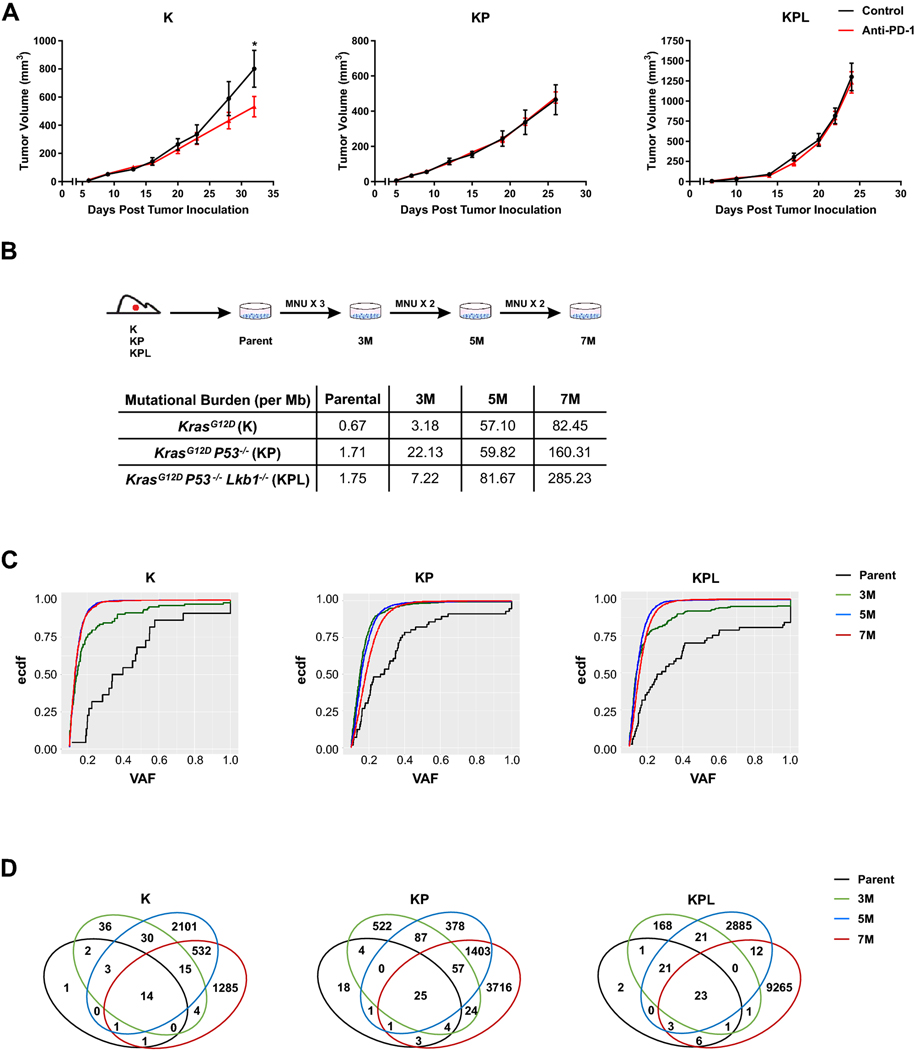
Murine models of NSCLC with varying mutational burden. **a** After subcutaneous (SC) tumor inoculation [K (2 × 10^6^) cells in 129-E mice; KP (8 × 10^5^) cells in FVB mice; KPL (1 × 10^5^) cells in FVB mice], mice bearing < 50mm^3^ tumors (∼ day 7) were treated with (i) isotype control, (ii) anti-PD-1 (200 μg/dose 3 times weekly for 4 doses), and tumor growth was measured with caliper. Results are representatives of at least two biological replicates of 6–10 mice per group. **b** K, KP, and KPL were exposed to 100 μg/mL of MNU for 45 min. Cells were passaged prior to additional exposures to MNU for a total of 3, 5, and 7 exposures (3M, 5M, 7M). TMBs revealed by WES analyses are shown in the table. **c** Empirical cumulative distribution function (ECDF) of the mutations is plotted against VAF as an illustration of tumor heterogeneity within each family of cells. **d** Venn diagram of shared and private mutations of the K, KP, and KPL isogenic cell lines. *P* values were determined by two-way ANOVA with Tukey post-test. *P < 0.05

Given the low mutational burden in *Kras*-mutant GEMMs of NSCLC, we reasoned these tumors would be resistant to anti-PD-1 therapy. We screened cell lines established from *Kras*-mutant GEMMs of NSCLC with common co-occurring genomic alterations, namely K, KP, and KPL, with anti-PD-1 antibody in immunocompetent mice. Primary resistance to anti-PD-1 immunotherapy was observed across all cell lines, except for K, which showed a statistically significant but modest response to PD-1 blockade (Fig. 1a, and Supplemental Figures S2A, S2B and S2C). Because loss of a functional PD-L1 axis has been implicated in primary resistance to ICIs, we assessed the capacity of IFN-γ to upregulate PD-L1 in K, KP, and KPL cells in vitro [15]. IFN-γ stimulation resulted in the upregulation of PD-L1 in all cell lines examined, confirming an intact PD-L1 axis (Supplemental Figure S2D).

To establish *Kras*-mutant NSCLC cell lines that harbor the essential genomic drivers of the disease and increased TMB, we subjected one cell line from each genomic background, namely K, KP and KPL, to the carcinogen MNU for various durations (3, 5, and 7 exposures to MNU for 45 min each time, designated as 3M, 5M, and 7M, respectively) (Fig. 1b). Although we evaluated other tobacco related carcinogens for this purpose, namely benzyo[a]pyrene and nicotine-derived nitrosamine ketone (NNK), we found MNU to have the highest in vitro efficacy to increase the mutational load of cell lines as determined by WES. MNU is a widely utilized in vivo carcinogen to generate murine models of NSCLCs [9]. Although the mechanism of MNU-mediated DNA damage is distinct from tobacco smoking, its potency and wide utility, documented in the literature, suggests MNU as an effective agent for generating passenger mutations in NSCLC cell lines that already possess the critical driver mutations of the *Kras*-mutant NSCLC. WES revealed a dose-dependent increase in the number of non-synonymous mutations across all genomic backgrounds, resulting in higher TMBs (Fig. 1b). In parallel to increased TMB within each family of isogeneic cell lines, we observed an increased proportion of mutations with low VAF, indicating increased tumor heterogeneity (Fig. 1c). MNU did not result in changes in the genomic copy number of parental cell lines which were diploid. Evaluation of the copy number and mutations of the driver genes confirmed the deletion *of Tp53* in KP cell lines and *Tp53* and *Lkb1* in KPL cell lines. Wild-type *Tp53* was preserved in K cell lines. No additional *Kra*s mutated alleles were detected in MNU-treated K, KP and KPL cell lines. The VAFs of the *Kras* mutation were around 0.4–0.6 in all cell lines, indicating that each cell line maintained the heterozygous *Kras* mutation. We then assessed the evolution of mutations within each family of cell lines with increased exposure to MNU, and detected mutations that were shared among parental, 3M, 5M and 7M cells within each genetic background, as well as private mutations which were specific to each cell line (Fig. 1d). The presence of both private and shared mutations in each family of cell lines provides a unique opportunity to evaluate the immune editing of these mutations in response to therapy in syngeneic studies.

### Higher TMB results in decreased tumor growth

**Fig. 2:**
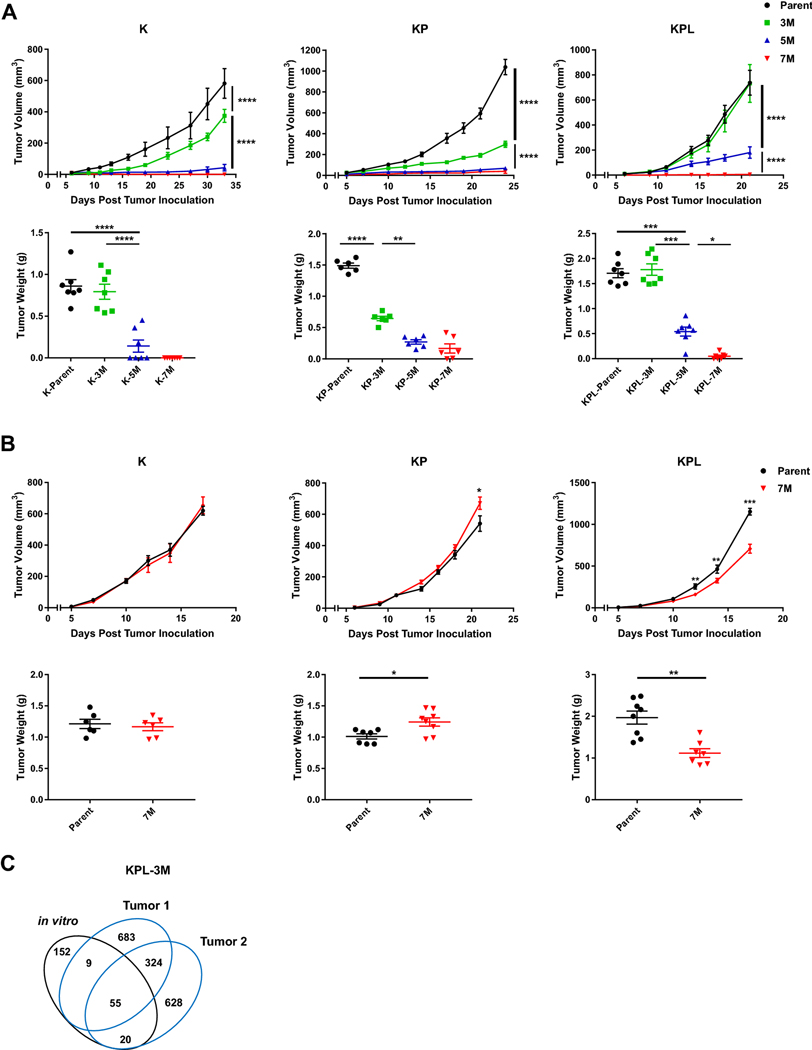
In vivo tumor growth in immunocompetent and SCID mice. **a** Within each family of cells, the Parent, 3M, 5M, and 7M cells were inoculated SC in immunocompetent mice [K (2 × 10^6^) cells in 129-E mice; KP (8 × 10^5^) cells in FVB mice; KPL (1 × 10^5^) cells in FVB mice] and tumor growth was measured with caliper. Growth curves and corresponding tumor weights after euthanasia are presented. **b** Same as in **a** except Parent and 7M cells were inoculated SC in SCID mice [K (2 × 10^6^) cells; KP (8 × 10^5^) cells; and KPL (1 × 10^5^) cells]. Data are representatives of at least two biological replicates of 6–10 mice per group. **c** Venn diagram of shared and private mutations of the KPL-3M cell line and two subcutaneous KPL-3M tumors from immunocompetent mice at day 25 post inoculation. *P* values were determined by two-tailed non-paired Student’s *t* test for pairwise comparison and two-way ANOVA with Tukey post-test for time-associated comparison among multiple groups. *P < 0.05; **P < 0.01; ***P < 0.001; ****P < 0.0001

To determine the effect of increased TMB on in vivo tumor growth, we screened each family of cell lines with varying TMB in syngeneic models. Across all genomic backgrounds, we observed diminished in vivo tumor growth with incremental increases in TMB (Fig. 2a). Increasing the number of injected cells led to robust tumor growth of K-3M, KP-3M, KPL-3M and KPL-5M cells, while other lines, namely K-5M, K-7M, KP-5M, KP-7M, KPL-7M, were rejected or displayed diminished growth rates (data not shown). Evaluation of in vitro growth rates of cell lines with varying TMB within each family revealed minimal differences (Supplemental Figure S3). These results indicate that diminished in vivo growth associated with increased TMB is likely immune-mediated. To further confirm this finding, we evaluated the in vivo growth of the K, KP and KPL parental cells, and their associated 7M counterparts in immunocompromised SCID mice which lack T and B cells (Fig. 2b). We observed similar tumor growth rates of K-7M and KP-7M compared to their parental counterparts, while KPL-7M showed minor but statistically significant reduction in tumor growth compared to parental KPL cells. Given that there was no difference in growth rates of KPL-P and KPL-7M in vitro (Supplemental Figure S3), the slightly reduced growth rate of KPL-7M in vivo could possibly be related to other immune-mediated pathways such as natural killer cells. These results suggest that the decreased tumor growth rates associated with high TMB in immunocompetent mice are predominantly due to host adaptive immune responses.

Next, we assessed the stability of the TMB in our syngeneic model system to ensure that in vivo host immune editing does not result in outgrowth of tumors with low TMB. We utilized KPL-3M as a representative model and performed WES of KPL-3M subcutaneous tumors in immunocompetent mice at day 25. We observed that subcutaneous KPL-3M tumors maintained 30% of the mutations present in KPL-3M cells prior to in vivo mouse inoculation (Fig. 2c). These represent stable mutations that are not edited by host immune cells. We also observed emergence of new mutations which likely result from the expansion of minor subclones that are below the detection threshold in KPL-3M cells (Fig. 2c). Overall, approximately 30% of total mutations were shared between two independent tumors (Fig. 2c). Taken together, these data suggest that the KPL-3M tumors maintain high TMB in vivo with a considerable overlap in their mutational profiles.

### High TMB is associated with increased local and systemic tumor-specific T cells

**Fig. 3:**
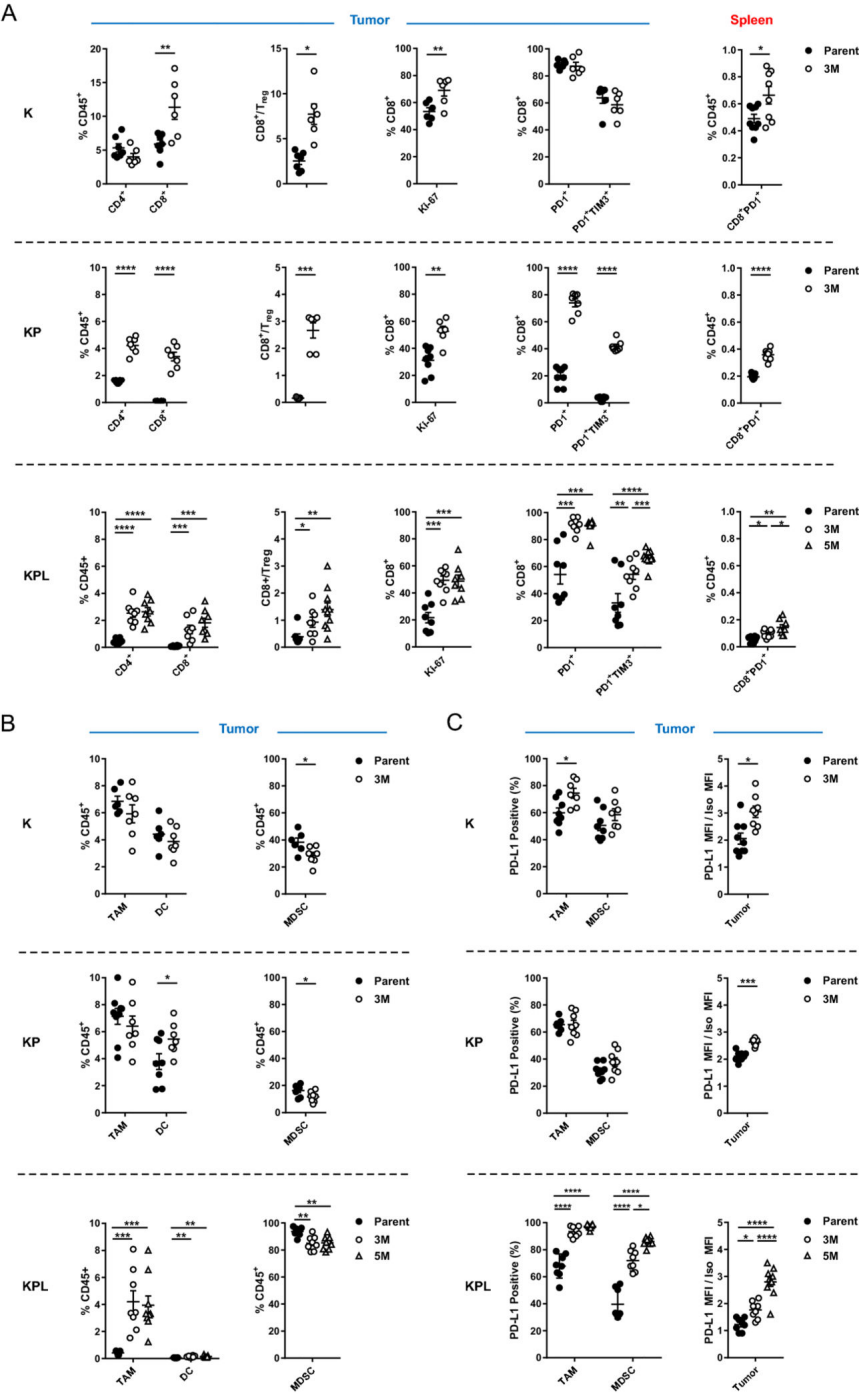
Distinct immune phenotypes of murine models revealed by FACS. On day 14–16 post-tumor inoculation [2 × 10^6^ K-Parent and K-3M cells in 129-E mice; 8 × 10^5^ KP-Parent and 2 × 10^6^ KP-3M cells in FVB mice; 1 × 10^5^ KPL-Parent, 1.5 × 10^5^ KPL-3M, and 3 × 10^5^ KPL-5M cells in FVB mice], tumors and spleens were harvested and analyzed by FACS. **a** Lymphoid compartment. **b** Myeloid compartment. **c** PD-L1 expression. Data are representatives of at least two biological replicates of 6–10 mice per group. *P* values were determined by *P* values were determined by two-tailed non-paired Student’s *t* test. *P < 0.05; **P < 0.01; ***P < 0.001; ****P < 0.0001

To define the immune responses induced by high TMB in each genetic background, we utilized cells with robust in vivo growth and moderate mutation burden, namely K-Parent, K-3M, KP-Parent, KP-3M, KPL-Parent, KPL-3M, and KPL-5M. We first evaluated the lymphoid compartment of the TME. We observed a significant increase in the number of tumor-infiltrating lymphocytes (TILs) and an increase in CD8^+^ to regulatory T (Treg) cell ratio in the TME with increased TMB in each genetic background (Fig. 3a). In addition, a higher percentage of infiltrating CD8^+^ T cells in the tumors with high TMB expressed the proliferation marker Ki-67 compared to their respective parental tumors.

Next, we evaluated the expression of the early activation/exhaustion marker PD-1 on TILs and observed higher expression of PD-1 on CD8^+^ T cells in KP and KPL tumors with high TMB compared to their parental counterparts (Fig. 3a). Studies reveal that tumor-specific CD8^+^ TILs in human cancers express high levels of PD-1 and that this phenotype can identify the diverse repertoire of clonally expanded tumor-reactive T cells. Thus, our results suggest that increased TMB in the KP and KPL models results in increased tumor-specific CD8^+^ T cell responses [16, 17]. In parallel, we detected increased co-expression of the checkpoint TIM-3, a marker of increased T cell exhaustion with prolonged antigen exposure, on PD-1^+^CD8^+^ T cells in KP and KPL tumors with high TMB compared to their parental counterparts. However, we observed no difference in tumor CD8 ^+^ T cell exhaustion between K-parent and K-3M tumors. This observation is likely due to increased immunogenicity of the K-parent tumors, which contain high TILs and increased CD8 ^+^ T cell exhaustion at baseline and exhibit slow in vivo growth with modest sensitivity to anti-PD-1 therapy. These results support the hypothesis that a higher TMB results in increased tumor-reactive PD-1^+^ T cells within the TME, which become exhausted with persistent antigen stimulation.

Peripheral tumor neoantigen-specific T cells which overlap with clonal tumor-specific TILs have also been identified in circulating PD-1^+^CD8^+^ T cells in melanoma patients [18]. Therefore, we evaluated the lymphoid compartment within the spleen of tumor-bearing mice, including expression of PD-1 on CD8 ^+^ T cells (Fig. 3a). We observed an increase in splenic PD-1^+^CD8^+^ T cells in mice bearing K, KP, and KPL tumors with high TMB compared to the parental counterparts, including a statistically significant increase in mice bearing KPL-5M tumors compared to those bearing KPL-3M tumors (Fig. 3a). These data suggest that increased TMB results in increased systemic tumor-specific T cells in our murine models.

Next, we evaluated the myeloid compartment of the TME and observed TMB-mediated changes in the immune phenotypes shared across all genetic backgrounds, as well as marked differences specific to KPL cells (Fig. 3b and Supplemental Figure S4A). High TMB was associated with a significant increase in the professional antigen-presenting dendritic cells (DCs) in the KP and KPL tumors, with no differences observed in K tumors. We observed no differences in the number of tumor-associated macrophages (TAMs) in K-3M and KP-3M compared to the respective parental tumors, but we observed an increase in TAMs in KPL-3M and KPL-5M tumors compared to KPL-Parent. We evaluated changes in the MDSCs and found that high TMB was associated with decreased MDSCs across all genetic backgrounds. KPL tumors contained a significantly higher percentage of MDSCs (over 80% of CD45^+^ cells), which were predominantly G-MDSCs expressing the neutrophil marker Ly6G (Fig. 3b and Supplemental Figure S4B). We observed a similar phenotype in the spleen where KPL tumor-bearing mice had a significantly higher percentage of G-MDSCs compared to mice bearing K and KP tumors (Supplemental Figure S4C). This immune phenotype in KPL models is consistent with studies in *Kras*-mutant murine and *KRAS*-mutant human NSCLC where LKB1 loss is associated with a T cell-suppressed and neutrophil-enriched TME [19–21].

We further evaluated the expression of PD-L1 by tumors and the myeloid cells in the TME (Fig. 3c). We observed an increase in PD-L1 expression on TAMs in K and KPL tumors with increased TMB compared to their parental counterparts but no difference between KP-3M and KP-Parent. We observed increased PD-L1 expression on MDSCs in the KPL tumors with higher TMB, with the greatest expression observed in KPL-5M, but no difference was detected within the K and KP genetic background. Increased PD-L1 expression was also observed on tumors with an increased TMB in each genetic background. Taken together, the observed overall trend of increased PD-L1 expression associated with increasing TMB implies amplified adaptive immune resistance within the TME.

### Anti-PD-1 responses in cell lines with high TMB recapitulate the therapeutic vulnerabilities of *KRAS*-mutant human NSCLC

**Fig. 4:**
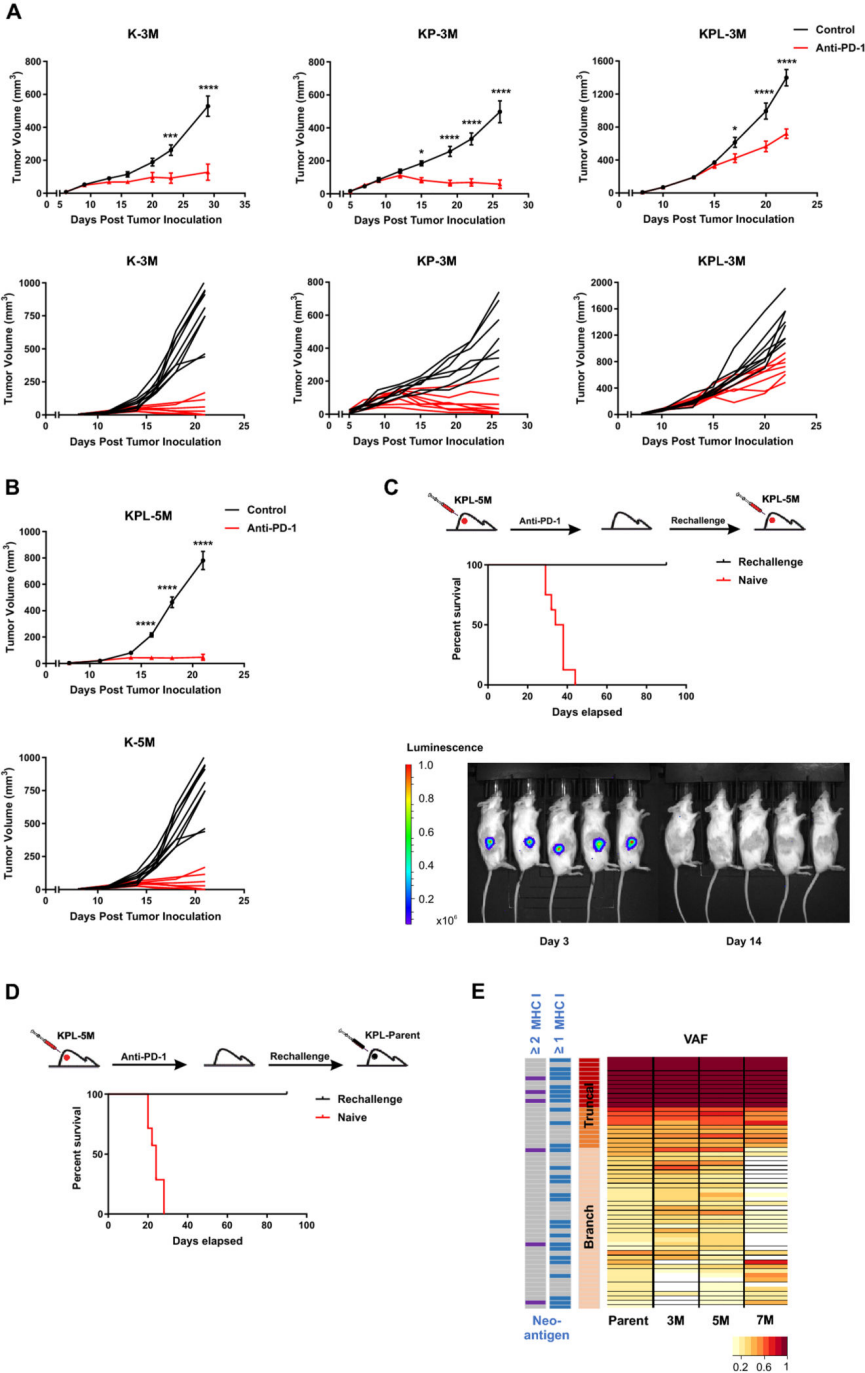
High TMB results in increased efficacy of anti-PD-1 therapy. **a** After SC tumor inoculation [K-3M (2 × 10^6^) cells in 129-E mice; KP-3M (2 × 10^6^) cells in FVB mice; KPL-3M (1.5 × 10^5^) cells in FVB mice], mice bearing < 50mm^3^ tumors (∼ day 7) were treated with (i) isotype control, (ii) anti-PD-1 (200 μg/dose 3 times weekly for 4 doses), and tumor growth was measured with caliper. Results are representatives of at least two biological replicates of 6–10 mice per group. **b** Same experimental design as **a** except that KPL-5M (3 × 10^5^) cells were utilized for SC tumor inoculation. **c** FVB naïve mice and mice that previously eradicated KPL-5M tumors in response to PD-1 blockade were inoculated SC with KPL-5M (3 × 10^5^) and tumor growth was measured with bioluminescence imaging on day 3 and day 14. Survival curve is presented. Data is representatives of two biological replicates of 5–6 mice per group. **d** FVB naïve mice and FVB mice that previously eradicated KPL-5M tumors in response to PD-1 blockade were inoculated SC with KPL-Parent (2 × 10^5^). Survival curve is presented. Data is representatives of two biological replicates of 5–6 mice per group. **e** Representation of the frequency of the mutations in the KPL-Parent tumors shared by KPL-3M, KPL-5M, or KPL-7M. The predicted neoantigens are presented based on MHC-I binding avidity by at least one or two MHC-I alleles (two left columns). *P* values were determined by two-way ANOVA with Tukey post-test. *P < 0.05; **P < 0.01; ***P < 0.001; ****P < 0.0001

We evaluated the efficacy of PD-1 blockade in K-3M, KP-3M and KPL-3M tumors with increased TMB (Fig. 4a and Supplemental Figure S5A). Anti-PD-1 therapy resulted in robust anti-tumor responses with an eradication of 33% of K-3M tumors. Similarly, 44% of KP-3M tumors were rejected and others stabilized in response to anti-PD-1. In contrast, anti-PD-1 efficacy was limited in KPL-3M tumors where PD-1 blockade resulted in reduced tumor growth without a complete rejection. This result is in agreement with the recent findings in human *KRAS*-mutant lung adenocarcinoma in which LKB1 loss was shown to be a major driver of primary resistance to PD-1 blockade [20]. We next assessed the efficacy of PD-1 blockade in mice bearing KPL-5M tumors and observed significant anti-tumor responses with the rejection of approximately 50% of tumors (Fig. 4b). These data suggest that the increased TMB of KPL-5M tumors could overcome the immunosuppressed TME and enhance responses to PD-1 blockade. This is in agreement with our immunophenotyping results of the KPL family of tumors in which mice bearing KPL-5M tumors possessed the highest number of local and systemic activated PD1^+^ CD8^+^ T cells (Fig. 3a). PD1^+^ CD8^+^ T cells have been shown to contain pools of tumor neoantigen-specific T cells that can be reinvigorated following PD-1 blockade [22, 23]. The mice bearing KPL-5M tumors, that had a complete anti-tumor response to anti-PD1, were subsequently rechallenged with KPL-5M cells. In response to the rechallenge, we observed an initial tumor growth followed by spontaneous rejection of all tumors (Fig. 4c), indicating the establishment of systemic anti-tumor immunity in response to PD-1 blockade in mice bearing KPL-5M tumors.

Given that KPL-5M shares 47 truncal and branch mutations with the KPL-Parent (Fig. 1d), we assessed whether the mice that had eradicated the KPL-5M tumors following anti-PD-1 treatment could reject the parental tumors by inoculating the mice with KPL-Parent cells 3 months after the initial rejection (Fig. 4d). All of the mice eliminated the KPL-Parent tumors after an initial growth, while the naïve control mice succumbed to implantation of KPL-Parent tumors in less than 30 days. Computational analysis of putative neoantigens in the KPL-Parent revealed 11 truncal and 17 branch neoantigens which were shared with KPL-3M, KPL-5M or KPL-7M (Fig. 4e and Supplemental Figure S5B). These results indicate the presence of tumor-specific memory T cells against shared neoantigen(s) between KPL-5M and KPL-Parent tumors in anti-PD-1 treated mice that had eradicated KPL-5M tumors. Despite this finding, T cell responses against these shared neoantigen(s) are not sufficient to eradicate KPL-Parent tumors in naïve mice treated with PD-1 blockade (Fig. 1a). This may be due to profound immunosuppression in the TME of KPL-Parent tumors that represses the initial host anti-tumor T cell responses (Fig. 3). In contrast, eradication of KPL-P tumors in the rechallenge experiments may be predominantly mediated by memory T cells, which can generate a rapid recall response to secondary challenge that overcomes the immunosuppressive TME. The presence of shared neoantigen(s) in these isogenic cell lines with varying TMB provides a unique opportunity to investigate immune responses against truncal and branch mutations in the context of TMB-associated changes in the TME.

In summary, we report novel *Kras*-mutant murine models of NSCLC bearing common driver gene alterations and increased TMB (Supplemental Table S2). Although the nature of the additional somatic mutations induced by MNU may not fully recapitulate the spectrum of tobacco-related mutations observed in human *KRAS*-mutant NSCLC, these murine models are clinically relevant because they possess the dominant driver mutations of *KRAS*-mutant NSCLC that determine distinct TMEs and clinical phenotypes as well as passenger mutations that can elicit adaptive immune responses. In contrast to existing *Kras*-mutant GEMMs of NSCLC that possess few mutations and have limited utility in immunotherapy studies, our models with increased TMB recapitulate the therapeutic vulnerabilities to anti-PD-1 which mirrors that of human *KRAS*-mutant NSCLC. The KPL-3M model with co-occurring *Kras* and *Lkb1* mutations, neutrophil-enriched TME, physiologically relevant TMB, and limited efficacy to anti-PD-1 serves as a clinically relevant model for preclinical immunotherapy studies, given loss of LKB1 is a dominant driver of resistance in human NSCLC. We anticipate that these novel immunogenic murine models will facilitate the development of future immunotherapies for NSCLC.

## Supplementary Material

1720556_Sup_info
