# Supplementary material for: Novel Kras-mutant murine models of non-small cell lung cancer possessing co-occurring oncogenic mutations and increased tumor mutational burden
Source: Cancer Immunol Immunother. 2021 Jan 28;70(8):2389–400. doi: 10.1007/s00262-020-02837-9 (PMC8289771; doi:10.1007/s00262-020-02837-9)
Supplement: Supplementary file 1 — Figure S1. Histological evaluation of the lung tissues 15 days after tail-vein injection of FVB mice with A) 1x10^5^ KP cells and B) 1x10^5^ KPL cells revealed poorly differentiated adenocarcinoma with marked nuclear atypia and high mitotic figures (x4 on the left and x20 on the right, hematoxylin and eosin stain). Figure S2. A) After SC tumor inoculation [K (2x10^6^) cells in 129-E mice; KP (8x10^5^) cells in FVB mice; KPL (1x10^5^) cells in FVB mice], mice bearing <50mm3 tumors (~day 7) were treated with i) isotype control, ii) Anti-PD-1 (200 μg/dose 3 times weekly for 4 doses). Tumor weights after euthanasia are presented. Data are representatives of at least two biological replicates of 6-10 mice per group. B) Same as A) except SC tumor inoculation of KPL-1950A (3x10^5^), KPL-1942B (1x10^6^), or KPL-2 (1x10^6^) cells in FBV mice. Growth curves and corresponding tumor weights after euthanasia are presented. C) Same as B) except SC tumor inoculation of KP-2042 (1.5x10^5^), KP-1.1 (2x10^6^), or KP-3.1 (2x10^6^) cells in FVB mice. D) In vitro PD-L1 expression of K, KP and KPL cells (triplicates) at baseline and after stimulation with IFN-γ at 100 ng/ml for 24 hours. P values were determined by two-tailed non-paired Student’s t test for pairwise comparison and two-way ANOVA with Tukey post-test for time-associated comparison among multiple groups. *, P<0.05. Figure S3. In vitro proliferation of K, KP and KPL family of cells by ATPlite in 8 replicates. Data at each time point is normalized to the reading at baseline to control for plating differences. P values were determined by two-way ANOVA with Tukey post-test. *, P<0.05; **, P< 0.01; ***, P<0.001; ****, P< 0.0001. Figure S4. On day 14-16 post-tumor inoculation [2x10^6^ K-Parent and K-3M cells in 129-E mice; 8x10^5^ KP-Parent and 2x10^6^ KP-3M cells in FVB mice; 1x10^5^ KPL-Parent, 1.5x10^5^ KPL-3M, and 3x10^5^ KPL-5M cells in FVB mice], tumors and spleens were harvested and analyzed by FACS. A) Flow gating strategy for the myeloid compartment. B) [file 262_2020_2837_MOESM1_ESM.pdf]

Table S1. FACS antibodies.

Immune Marker	Fluorophore	Clone
CD45	perCP/Cy5.5	30-F11
CD3	AF700	17A2
CD8	BV421	53-6.7
CD4	BV650	GK1.5
CD25	APC	PC61
FOXP3	PE	150D
PD1	PE-Cy7	29F.1A12
Tim3	PE	RMT3-23
Ki67	BV605	16A8
CD11c	FITC	HL3
IA/IE	BV650	M5/114.15.2
CD11b	AF700	M1/70
Ly6C	APC	HK1.4
Ly6G	PE	1A8
CD64	PE-Cy7	X54-5/7.1
PD-L1	BV421	29E.2A3
Zombie NIR		

Fig S1

A

KP

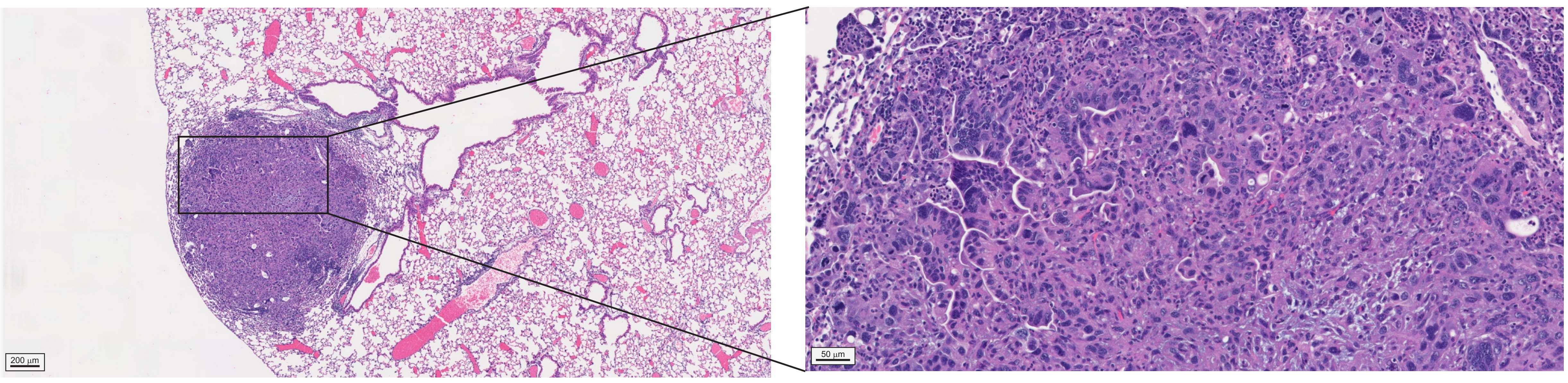

B

KPL

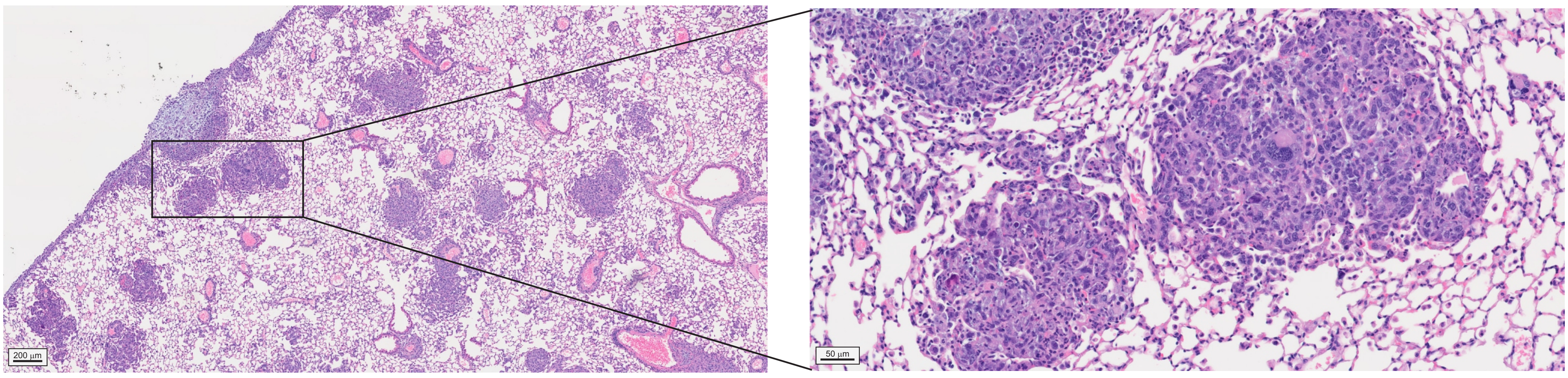

Fig S2

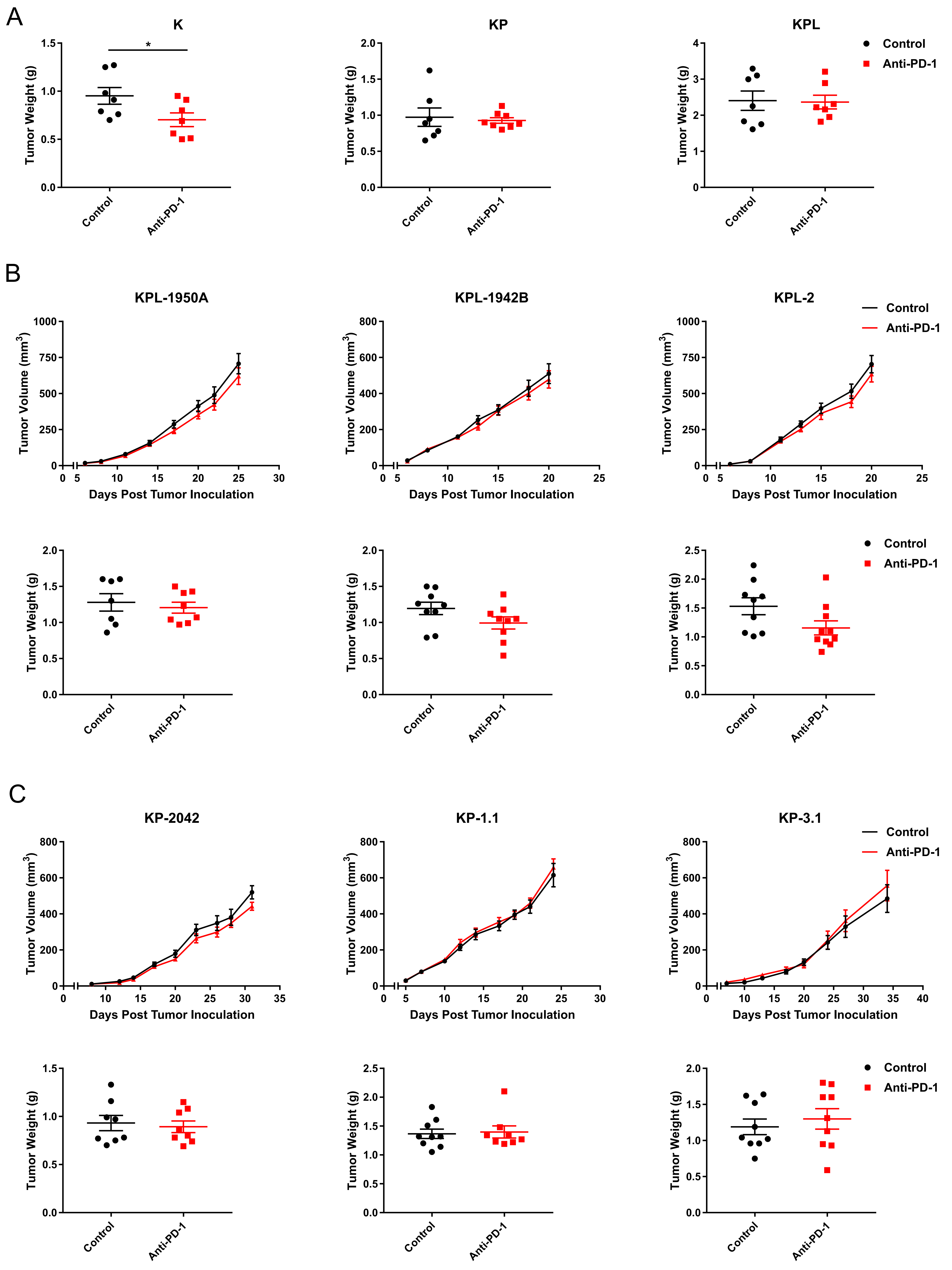
A
K
KP
KPL
Control
Anti-PD-1
Tumor Weight (g)
*
B
KPL-1950A
KPL-1942B
KPL-2
Tumor Volume (mm3)
Days Post Tumor Inoculation
C
KP-2042
KP-1.1
KP-3.1

Fig S2

D

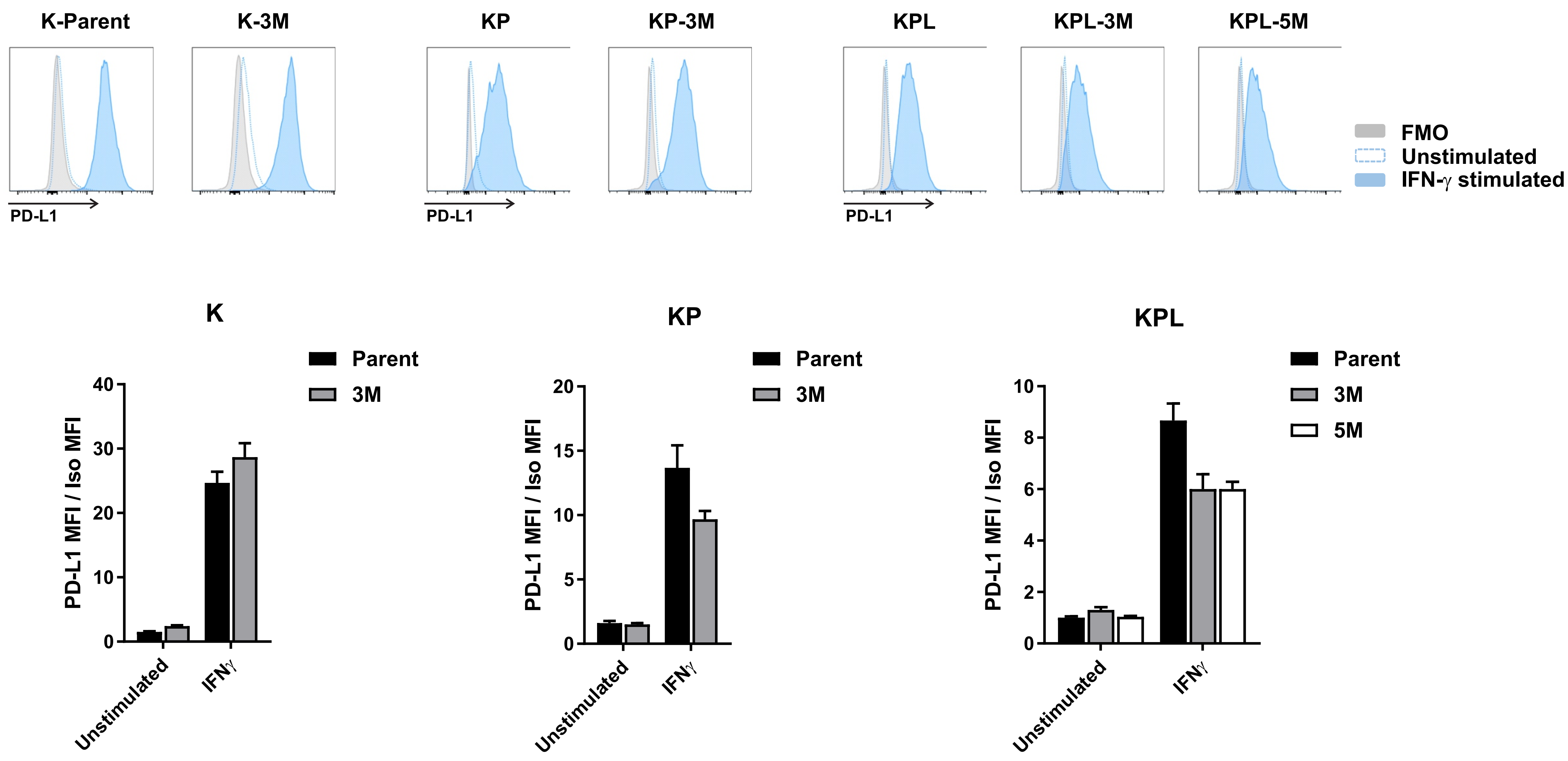
K-Parent
K-3M
KP
KP-3M
KPL
KPL-3M
KPL-5M
PD-L1
FMO
Unstimulated
IFN-γ stimulated
K
KP
KPL
Parent
3M
5M
PD-L1 MFI / Iso MFI
Unstimulated
IFNγ
0
10
20
30
40
5
15
2
4
6
8

Fig S3

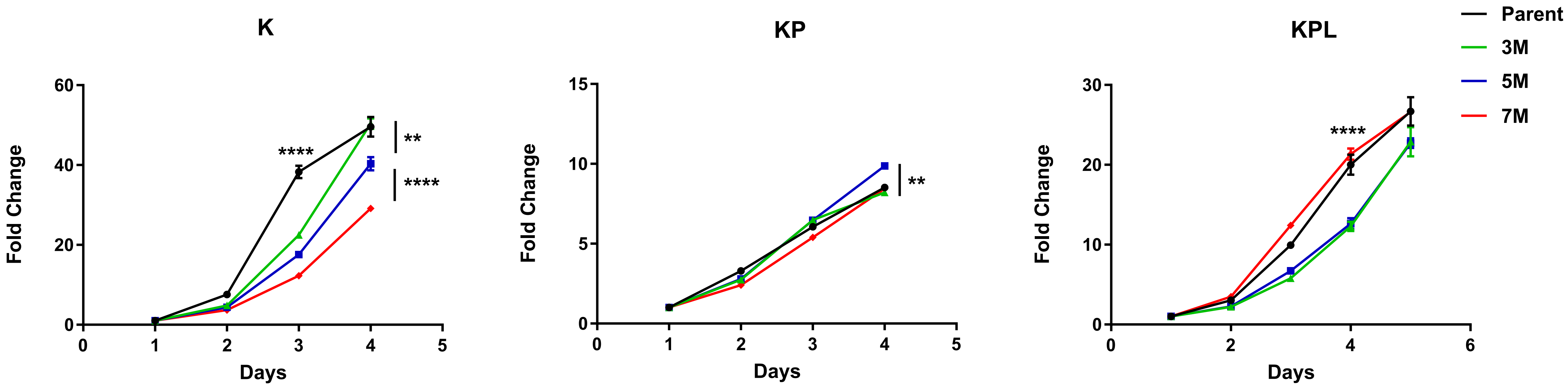

Fig S4

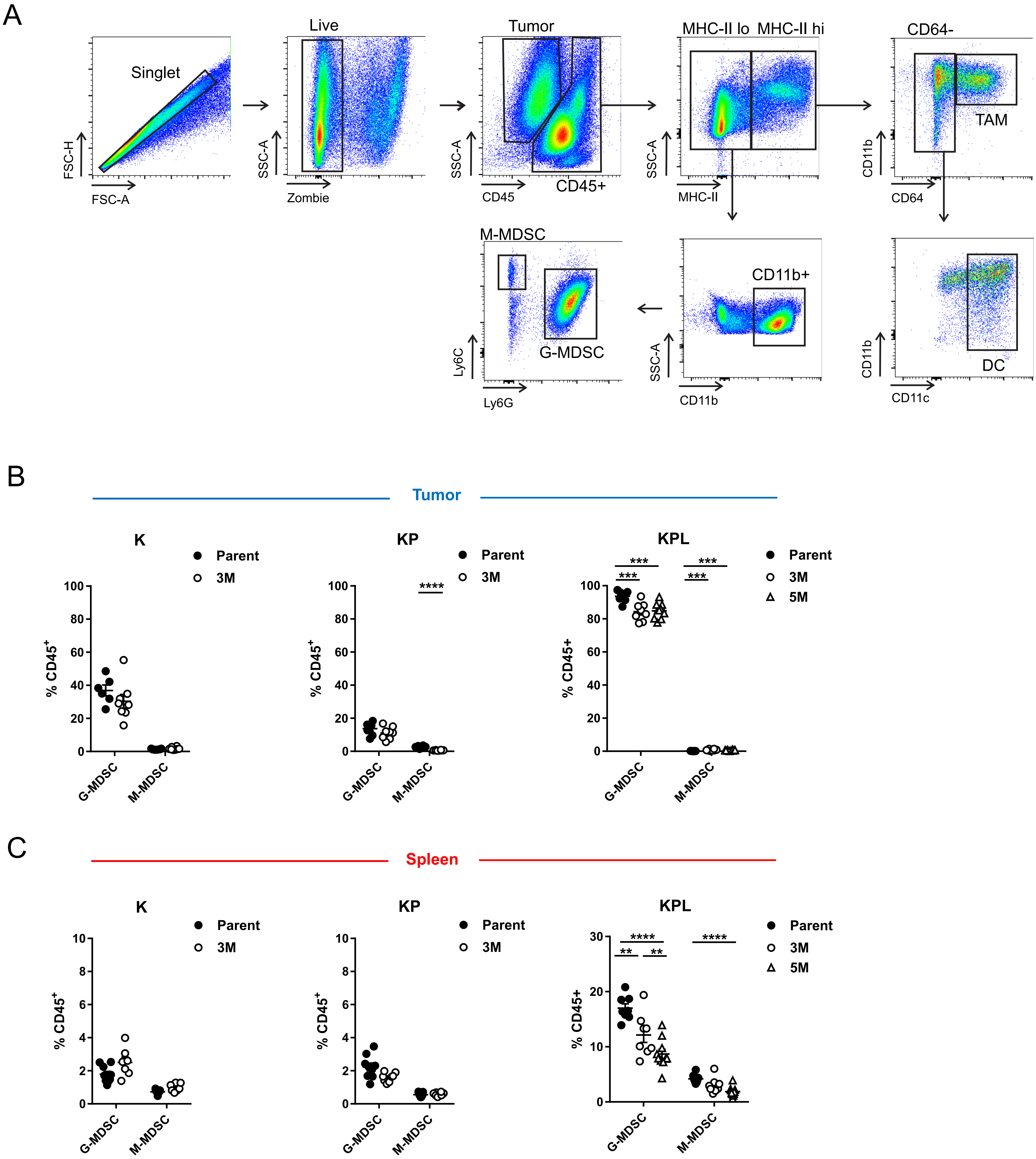
A
Singlet
FSC-H
FSC-A
Live
SSC-A
Zombie
Tumor
CD45
CD45+
MHC-II lo
MHC-II hi
MHC-II
CD64-
CD11b
CD64
TAM
M-MDSC
G-MDSC
Ly6C
Ly6G
CD11b+
CD11c
DC
B
Tumor
K
KP
KPL
Parent
3M
5M
% CD45+
G-MDSC
M-MDSC
C
Spleen

Fig S5

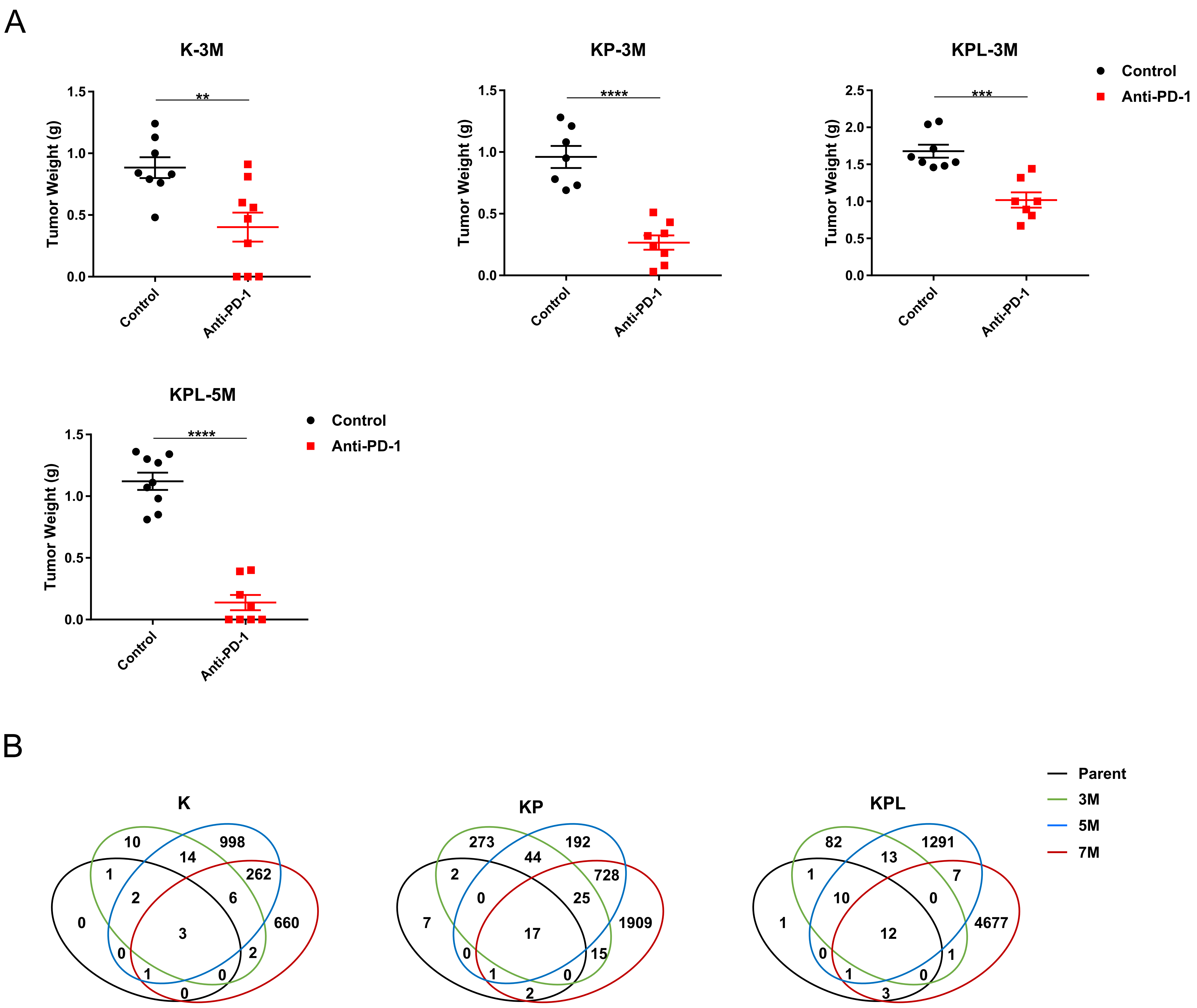
A
K-3M
KP-3M
KPL-3M
KPL-5M
Tumor Weight (g)
Control
Anti-PD-1
**
****
***
****
B
K
KP
KPL
Parent
3M
5M
7M

Table S2. Summary of Key Characteristics of *Kras*-Mutant NSCLC Cell Lines.

Genetic background	Cell ID	TMB (per Mb)	TME (% of immune cells)					Anti-PD-1 Efficacy
			CD4	CD8	G-MDSC	TAM	DC	
Kras^{G12D} (K)	Parent	0.67	5%	6%	38%	7%	4%	Modest
	3M	3.18	4%	11%	32%	6%	4%	Robust
Kras^{G12D}*P53*$^{-/-}$ (KP)	Parent	1.71	2%	<1%	15%	7%	4%	Resistant
	3M	22.13	4%	3%	14%	6%	5%	Robust
Kras^{G12D}*P53*$^{-/-}$*LKB1*$^{-/-}$ (KPL)	Parent	1.75	<1%	<1%	95%	<1%	<1%	Resistant
	3M	7.22	2%	1%	84%	4%	<1%	Modest
	5M	81.67	3%	2%	84%	4%	<1%	Robust
